# GABAergic currents in RT and VB thalamic nuclei follow kinetic pattern of α3- and α1-subunit-containing GABA_A_ receptors

**DOI:** 10.1111/j.1460-9568.2007.05693.x

**Published:** 2007-08

**Authors:** Jerzy W. Mozrzymas, Andrea Barberis, Stefano Vicini

**Affiliations:** 1 Department of Physiology and Biophysics, Georgetown University School of Medicine Washington, DC 20007, USA; 2 Laboratory of Neuroscience, Department of Biophysics, Wroclaw Medical University Chalubinskiego 3, 50–368 Wroclaw, Poland

**Keywords:** GABA, IPSC, kinetics, rat

## Abstract

Inhibitory postsynaptic currents (IPSCs) of the thalamic reticular (RT) nucleus are dramatically slower than in the neighboring ventrobasal (VB) neurons. It has been suggested that α3-subunit-containing receptors underlie slow IPSCs in RT neurons, while rapid synaptic currents in the VB nucleus are due to γ-aminobutyric acid A receptors (GABA_A_Rs), including the α1-subunit. In our recent study [Barberis *et al.* (2007) *Eur. J. Neurosci*., **25**, 2726–2740] we have found that profound differences in kinetics of currents mediated by α3β2γ2 and α1β2γ3 receptors resulted from distinct binding and desensitization properties. However, a direct comparison between kinetics of neuronal GABA_A_Rs from RT and VB neurons and α3- and α1-subunit-containing receptors has not been made. For this purpose, current responses to ultrafast GABA applications were recorded from patches excised from neurons in VB and RT areas. Deactivation kinetics determined for RT and VB neurons closely resembled that in currents mediated by α3β2γ2 and α1β2γ2 receptors. In RT neurons, currents elicited by non-saturating [GABA] had a remarkably slow onset, a hallmark of α3-subunit-containing receptors. In VB and RT neurons, single-channel currents elicited by brief GABA pulses had similar characteristics to those of α1β2γ2 and α3β2γ2 receptors. However, in stationary conditions, similarity between single-channel currents in neurons and respective recombinant receptors was less apparent. We propose that the non-stationary kinetics of GABAergic currents in VB and RT nuclei mimic that of currents mediated by α1- and α3-subunit-containing receptors. The dissimilarity between stationary kinetics of neuronal and recombinant receptors probably reflects differences between GABA_A_Rs mediating phasic and tonic currents in these neurons.

## Introduction

Thalamic reticular (RT) inhibitory γ-aminobutyric acid (GABA)ergic neurons and thalamocortical excitatory ventrobasal (VB) neurons are fundamental in regulating sensory gating, sleep patterns, and are involved in the mechanism of absence epilepsy (Steriade *et al.*, 1993; von Krosigk *et al.*, 1993; Lee *et al.*, 1994; Huguenard, 1999; Blethyn *et al.*, 2006). The inhibitory synaptic currents (IPSCs) in the RT neurons are the slowest in the CNS (Huntsman & Huguenard, 2000, 2006; Browne *et al.*, 2001), while in neighboring VB neurons IPSCs are markedly faster and undergo a developmental speed up (Huntsman & Huguenard, 2000; Okada *et al.*, 2000; Browne *et al.*, 2001), as in the rest of the brain (Takahashi, 2005). Interestingly, the distinct IPSC kinetics in these nuclei are correlated with differential expression of GABA_A_ receptor (GABA_A_R) subunits (Fritschy & Mohler, 1995; Pirker *et al.*, 2000; Browne *et al.*, 2001; Kralic *et al.*, 2006). In the RT nucleus, the α3-subunit is abundant during development and in adulthood (Laurie *et al.*, 1992; Pirker *et al.*, 2000; Kralic *et al.*, 2006), and is not affected by deletion of the α1-subunit (Kralic *et al.*, 2006). In contrast, during early development, the predominant α2-subunit in VB is replaced by the α1-subunit (Laurie *et al.*, 1992; Fritschy *et al.*, 1994). Moreover, in VB neurons, α4- and δ-subunits are expressed (Laurie *et al.*, 1992; Chandra *et al.*, 2006; Kralic *et al.*, 2006), giving rise to a tonic conductance (Belelli *et al.*, 2005; Cope *et al.*, 2005; Jia *et al.*, 2005; Chandra *et al.*, 2006), a molecular target for hypnotics (Belelli *et al.*, 2005).

Browne *et al.* (2001) suggested that the kinetic properties of GABA_A_Rs in RT neurons were underlying slow IPSC, and single-cell polymerase chain reaction indicated the involvement of the α-subunit, possibly α1/α2 vs α3, as the structural determinant of channel kinetics in VB and RT neurons. In our recent study (Barberis *et al.*, 2007) we have reported that α3β2γ2 receptors had the longer mean burst length by about 60% than α1β2γ2 receptors, while deactivation kinetics mediated by these receptors were several fold different (Verdoorn, 1994; Gingrich *et al.*, 1995; Barberis *et al.*, 2007). Analysis of current responses to ultrafast GABA applications revealed that these receptors substantially differed in binding and gating (Barberis *et al.*, 2007). It seems interesting to examine to what extent the GABA_A_R kinetics in VB and RT nuclei are compatible with kinetic patterns of α1β2γ2 and α3β2γ3 receptors. To this end, current responses to rapid GABA applications were recorded from VB and RT nuclei. Deactivation kinetics determined for VB and RT neurons closely resembled those observed for α1β2γ2 and α3β2γ2 receptors. Moreover, in RT neurons, currents elicited by non-saturating [GABA] had a remarkably slow onset, a hallmark of α3-subunit-containing receptors. While single-channel currents evoked by brief GABA pulses in VB and RT neurons resembled those observed for α1β2γ2 and α3β2γ2 receptors, in steady-state conditions such a similarity was less apparent. This might reflect differences between receptors mediating phasic and tonic GABAergic currents.

## Materials and methods

### Slice preparation

Postnatal day (P)14–19 mice were killed by decapitation in agreement with the guidelines of the Georgetown University Animal Care and Use and the Polish Animal Welfare Act. Slices were prepared from P14–19 C57BL/6J, strain 129/Sv/SvJ mice (Taconic). Whole brains were dissected and placed in an ice-cold slicing solution containing (in mm): NaCl, 85; KCl, 2.5; CaCl_2_, 1; MgCl_2_, 4; NaH_2_PO_4_, 1; NaHCO_3_, 25; glucose, 25; sucrose, 75 (all from Sigma); pH 7.4 when continuously bubbled with 95% O_2_ and 5% CO_2_. Coronal slices (200–300 µm) were prepared using the Vibratome 3000 Plus Sectioning System (Vibratome, St Louis, MO, USA) or VT 1000S (Leica, Nussloch, Germany). These slices included the thalamus and the internal capsule, cortex and hippocampus. Slices were incubated in the slicing solution at 34 °C for 30 min before being transferred to the recording solution. Slices were viewed under an upright microscope (E600FN, Nikon) equipped with Nomarski optics and an electrically insulated 60 × water immersion objective with a long working distance (2 mm) and high numerical aperture (1.0), or with a DM LFSA upright microscope (Leica Microsystems, Wetzler, Germany).

### Electrophysiological recordings

Electrophysiological recordings were performed either in the whole-cell (IPSC measurements) or in the outside-out configuration (current responses to exogenous GABA) of the patch-clamp technique at a pipette voltage of −70 mV using the Axopatch 200B amplifier (Molecular Device, Sunnyvale, CA, USA) or the Multiclamp 700B amplifier (Molecular Device, Union City, CA, USA). All the electrophysiological experiments were performed at room temperature (22–24 °C). Slices were perfused with an extracellular solution composed of (in mm): NaCl, 120; KCl, 3.1; K_2_HPO_4_, 1.25; NaHCO_3_, 26; dextrose, 5.0; MgCl_2_, 1.0; CaCl_2_, 2.0; with the addition of 1 µm tetrodotoxin (Sigma, St Louis, MO, USA). The solution was maintained at pH 7.4 by bubbling with 5% CO_2_ + 95% O_2_. Patch pipettes were filled with internal solution containing (in mm): CsCl, 145; CaCl_2_, 1; 1,2-bis(2-aminophenoxy)ethane-*N*,*N*,*N*′-tetraacetic acid (BAPTA), 11; MgATP, 4; HEPES, 10 (pH 7.2 with CsOH). For IPSC recordings in the whole-cell patch-clamp mode, patch electrodes of 3.5–5 MΩ (when filled with internal solution) were pulled with a vertical puller (Narishige, PP-83, Tokyo, Japan) from borosilicate glass capillary (Drummond, Broomall, PA, USA). Excised patches were pulled after the whole-cell recordings of IPSCs. However, a better stability of recordings in the excised-patch configuration was achieved with pipettes with a higher resistance (5–7 MΩ), and part of the recordings from excised patches was made using electrodes with higher resistance. Recordings of current responses in human embryonic kidney (HEK) cells were performed in the excised-patch configuration. For HEK cells a HEPES-buffered external solution was used: (in mm): NaCl, 145; KCl, 5; CaCl_2_, 1; MgCl_2_, 1; glucose, 5; HEPES, 5 (pH 7.4 with NaOH).

Current signals collected in the whole-cell mode (IPSCs and firing pattern) were filtered at 3 kHz with an 8-pole Bessel filter, and sampled at 20 kHz using the analog-to-digital converter Digidata 1322A (Axon Instruments) and stored on the computer hard disk. For the analysis requiring a high temporal resolution (e.g. rise time kinetics of evoked currents), the signals were low-pass filtered at 10 kHz and sampled at 50–125 kHz. pClamp 9.2 (Molecular Device) software was used for acquisition and data analysis.

### Drug application

GABA-containing solution was applied to excised patches using an ultrafast perfusion system based on piezoelectric-driven theta-glass application pipette (Jonas, 1995). The piezoelectric translator used was the P-245.30 Stacked Translator (Physik Instrumente, Waldbronn, Germany), and theta glass tubing was from Hilgenberg (Malsfeld, Germany). The open-tip recordings of the liquid junction potentials revealed that the 10–90% exchange of solution occurred within 60–100 µs. The speed of the solution exchange was also estimated around the excised patch by the 10–90% onset of the membrane depolarization induced by application of high (25 mm) potassium saline. In this case the 10–90% rise time value (70–120 µs) was very close to that found for the open-tip recordings. Amplitude and deactivation kinetics were within 5% of the initial values in most experiments. Recordings with greater changes were discarded.

### Analysis

The decaying phase of the currents was fitted with a function in the form: *y*(*t*) *=* Σ*A*_*i*_ exp(*–t*/τ_*i*_), where *A*_*i*_ are the fractions of respective components (Σ*A*_*i*_ = 1) and τ_*i*_ are the time constants. The deactivation time course was well fitted with a sum of two exponentials (*n* = 2, see Results). The averaged deactivation time constant τ_m_ was calculated using the formula: τ_m_ = Σ*A*_*i*_τ_*i*_. The time course of desensitization onset was described by using a sum of an exponential function and a constant value representing the steady-state current during a continuous application of saturating [GABA].

Single-channel analysis was performed as in Barberis *et al.* (2007). Data are expressed as mean ± SEM, and unpaired Student's *t*-tests were used for data comparison.

## Results

### Miniature (m)IPSCs recorded from VB and RT nuclei show profoundly different kinetics

**Fig. 1:**
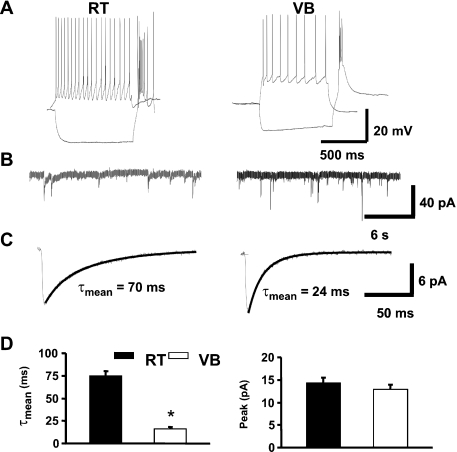
Thalamic reticular (RT) and ventrobasal (VB) neurons show distinct firing pattern and mIPSC kinetics. (A) Typical examples of firing pattern recorded in response to depolarizing (upper traces) and hyperpolarizing (lower traces) current stimulus in RT (left) and VB neurons (right). (B) Examples of mIPSC recordings at −70 mV in RT (left) and in VB neurons (right). (C) Averaged mIPSCs together with biexponential fits superimposed on the decaying current phases for RT (left) and VB neurons (right). (D) Summary of data derived for the assessment of the averaged decay time constant (τ_mean_) and peak mIPSCs amplitudes. *P < 0.05 indicates a significant difference.

mIPSCs were recorded from neurons localized either in VB or RT nuclei. Prior to mIPSC recordings, firing patterns were recorded (1 s of hyperpolarizing current, 1 s of zero current, 1 s of depolarizing current, 100–250 pA current depending on input resistance). As shown in Fig. 1A, in the VB nucleus the hyperpolarization showed a characteristic maximum suggesting activation of a hyperpolarization-activated (I_H_-like) conductance (Sun *et al.*, 2003). Such a phenomenon was never observed in RT neurons (Fig. 1A). In neurons from both nuclei, release of hyperpolarizing current resulted in a depolarization (probably due to ‘T’-type calcium channels) associated with the appearance of action potentials (Fig. 1A). Application of depolarizing current elicited a train of slowly adapting action potentials in neurons from both nuclei (Fig. 1A), but in the RT nucleus the spikes had a higher frequency. These distinct firing patterns were consistently observed and, together with anatomical localization, were applied as a criterion to select neurons from RT and VB nuclei.

Standard whole-cell recordings of mIPSCs were performed from neurons in VB and RT nuclei at the membrane voltage of −70 mV. We found that decaying phases of mIPSCs in RT nucleus were slower than those recorded in VB neurons (τ_mean_ = 74.7 ± 5.4, *n* = 11; and 16.5 ± 1.8, *n* = 14, for RT and VB, respectively, Fig. 1B). The mean mIPSC amplitudes measured in VB and RT neurons did not show significant differences (Fig. 1D). This observation confirms that in our experimental conditions decay of GABAergic synaptic currents is slower in RT neurons than VB neurons (Huntsman & Huguenard, 2000; Browne *et al.*, 2001).

### Deactivation of currents recorded from VB and RT nuclei show profound differences

**Fig. 2:**
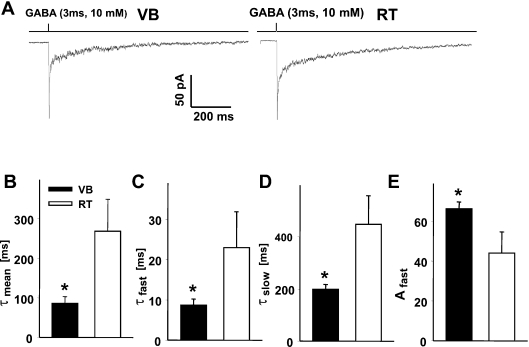
Deactivation kinetics in thalamic reticular (RT) and ventrobasal (VB) neurons show profound difference. (A) Typical examples of normalized current responses elicited by brief applications of saturating γ-aminobutyric acid (GABA; 3 ms, 10 mm). (B–E) Summary of data for the assessment of the averaged decay time constant (τ_mean_, B), fast deactivation component (τ_fast_, C), slow deactivation component (τ_slow_, D), and percentage of the fast component (*A*_fast_, E) *P < 0.05 indicates a significant difference.

GABA transient in a GABAergic synapse is known to be extremely brief (Clements, 1996; Overstreet *et al.*, 2002; Mozrzymas *et al.*, 2003a; Mozrzymas, 2004), and therefore the time course of synaptic currents is believed to largely represent the deactivation process (current time course following removal of agonist). Because profoundly different IPSC decay kinetics in neurons from VB and RT nuclei most likely result from differences in receptor gating, it is interesting to investigate the deactivation kinetics of currents elicited by exogenous agonist applications to the patches excised from neurons in these nuclei. To this end, an ultrafast perfusion system was used to evoke current responses to brief and saturating (3 ms, 10 mm) GABA pulses. The amplitudes of currents recorded from both thalamic areas showed large patch to patch variability, ranging from a few pA up to several hundred pA. The averaged value of current amplitude was larger in patches from VB (−97 ± 29 pA, *n* = 15) than in patches from RT neurons (−65 ± 29 pA, *n* = 9), but due to large scatter of these values this difference did not reach statistical significance. Currents recorded from patches excised from VB and RT neurons showed clearly different kinetics (Fig. 2A). In patches from neurons in both areas, the deactivation time course could be fitted well with two exponential components, although in the case of large currents a third component could be detected. However, as in most responses (especially those with low amplitude) such third (slowest) component could be hardly resolved from the baseline noise, a two exponential fit was used to describe the deactivation kinetics in neurons from both areas. The deactivation time course was clearly slower in currents recorded from patches from RT cells (Fig. 2A and B), and the averaged decay time constants were 268 ± 80 ms (*n* = 5) and 86 ± 17 ms (*n* = 16) in patches from RT and VB neurons, respectively. Both time constants of deactivation were significantly larger in currents recorded in patches from RT neurons (in VB τ_fast_ = 8.7 ± 1.5 ms, τ_slow_ = 199 ± 19 ms, *n* = 16; and in RT τ_fast_ = 23.0 ± 8.8 ms, τ_slow_ = 447 ± 110 ms, *n* = 5, Fig. 2C and D). Moreover, the percentage of the fast component was significantly larger in currents recorded from VB neurons (0.66 ± 0.03, *n* = 16 and 0.44 ± 0.1, *n* = 5 for VB and RT neurons, respectively, Fig. 2E).

Importantly, the pattern of deactivation kinetics in patches from VB and RT neurons is largely reproduced in currents recorded from patches from HEK cells expressing α1β2γ2 and α3β2γ2 receptors. As reported by Barberis *et al.* (2007), averaged deactivation time constants (τ_mean_) determined for currents mediated by α1β2γ2 and α3β2γ2 receptors were 52.5 ± 2.9 ms and 221.35 ± 14.9 ms, respectively, similar to those found for currents recorded from VB and RT neurons.

These findings further indicate that a profound difference in the mIPSC deactivation kinetics in VB and RT nuclei is associated with differences in gating properties of GABA_A_Rs expressed in neurons from these nuclei. This result is compatible with the previous molecular biology data (see Introduction) that indicated a correlation between kinetics of IPSCs and differential expression GABA_A_R subtypes in these nuclei.

### In RT neurons current responses to non-saturating GABA concentration show markedly slower onset than in VB cells

**Fig. 3:**
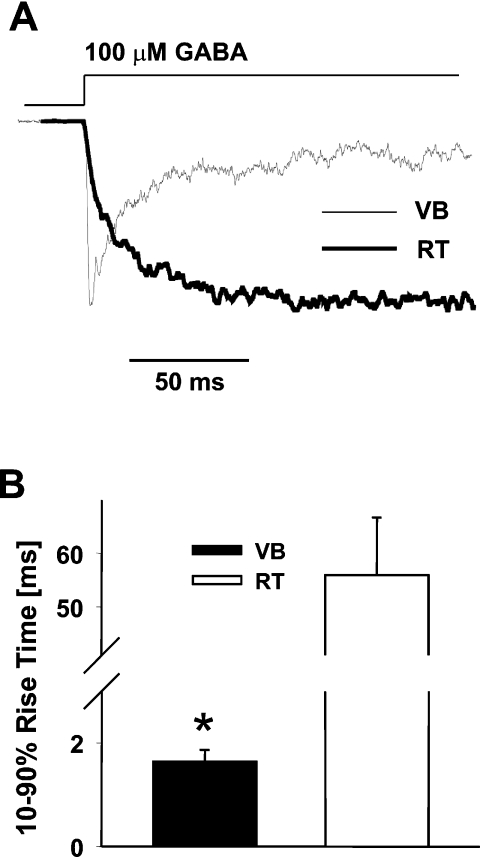
The onset kinetics of currents evoked by non-saturating γ-aminobutyric acid [GABA] in thalamic reticular (RT) is much slower than in ventrobasal (VB) neurons. (A) Typical examples of normalized current responses elicited by applications of 100 µm GABA in VB (thin line) and in RT neurons (thick line). (B) Summary of data for the assessment of the averaged values of 10–90% rise times for currents recorded from VB and RT neurons. *P < 0.05 indicates a significant difference.

While at saturating GABA concentrations, the binding step occurs much faster than the transitions between the bound states, at lower non-saturating [GABA] the rate of receptor activation is expected to strongly depend on the receptor's binding kinetics. Thus, to assess the difference in the binding step, current responses were recorded for 100 µm GABA, a concentration that is known to be non-saturating (especially for the onset kinetics, Mozrzymas *et al.*, 2003b) for most GABA_A_R subtypes. As shown in Fig. 3, the 10–90% rise time was several fold slower in RT neurons (1.64 ± 0.23 ms, *n* = 4, and 55.9 ± 10.8 ms, *n* = 7, in VB and RT, respectively, *P* < 0.05). While the values of rise times in patches from VB neurons were quite homogeneous (between 1 and 2 ms), the 10–90% rise times in RT cells showed considerably larger scatter (35–98 ms). Importantly, the difference in 10–90% rise time in patches from VB and RT neurons was similar to that observed for currents mediated by α1β2γ2 and α3β2γ2 receptors. Indeed, as reported in Barberis *et al.* (2007), 10–90% rise times measured in these experiments showed a dramatic difference: 2.1 ± 0.40 ms and 96.2 ± 8.8 ms for patches from α1β2γ2- and α3β2γ2-expressing cells, respectively.

These data demonstrate that the onset kinetics of currents elicited by a non-saturating GABA in patches from VB and RT neurons can be reasonably reproduced by currents mediated by α1- and α3-subunit-containing receptors. The fact that in patches from RT neurons 10–90% rise time showed a considerable scatter and faster averaged 10–90% rise time than in the case of α3β2γ2-mediated currents might suggest that in RT neurons, besides α3-subunit-containing receptors, there could be a contribution from other receptor subtypes.

### Single-channel properties in VB and RT neurons during the deactivation phase show different properties

**Fig. 4:**
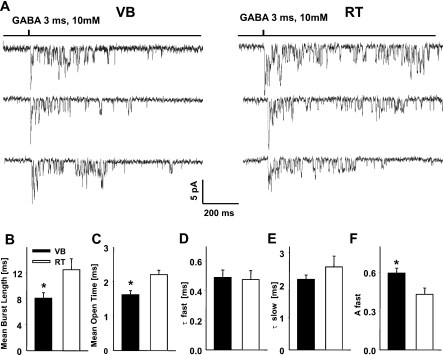
Non-stationary single-channel behavior during the deactivation phase reveals differences in γ-aminobutyric acid receptor (GABA_A_R) kinetics in neurons from ventrobasal (VB) and thalamic reticular (RT) regions. (A) Typical example of multiple records illustrating single-channel activity evoked by 3 ms applications of 10 mm GABA to outside-out patches in a VB (left panel) and a RT neuron (right panel). (B–F) Summary of data for the assessment of the mean burst length (B), mean open time (C), fast component of the open time distribution (τ_fast_, D), slow component of the open time distribution (τ_slow_, E) and percentage of the fast component (*A*_fast_, F) *P < 0.05 indicates a significant difference.

It is interesting how differences in macroscopic deactivation kinetics of GABA-evoked currents in patches from VB and RT neurons are correlated with the single-channel properties in the same experimental conditions. As shown in Fig. 4, in patches containing few channels it was possible to resolve single-channel activity during the decaying phase of responses to short and saturating GABA pulses (Fig. 4A). In both types of neurons, bursts were separated by silent periods (Fig. 4A), indicating that the deactivation time course represents a complex behavior including sojourns in an inactive fully bound (most likely desensitized, Jones & Westbrook, 1995) state. The closed time distributions determined for currents measured in patches from VB and RT areas could be fitted with at least three exponential components (not shown). For recordings from neurons in VB and RT, in all considered distributions, two shortest components (tenths of milliseconds and approximately 1–2 ms) were relatively constant and much shorter with respect to the slower components. Moreover, the slower time constants showed substantial variability depending on the number of channels in the patch. Thus, the two shortest components were interpreted as intraburst closures, and the critical time (*T*_crit_) was calculated by equalizing the proportion of misclassifications using the formula: 1 − exp(–*T*_crit_/τ_burst_) = exp(–*T*_crit_/τ_extraburst_), according to Colquhoun & Sakmann (1985). The averaged burst length was significantly longer in RT neurons (8.1 ± 0.8 ms, *n* = 10, and 12.5 ± 1.7 ms, *n* = 5, in VB and RT, respectively, *P* < 0.05, Fig. 4B). The mean intraburst interval as well as total mean open time were significantly shorter in patches from VB cells (in VB: 1.84 ± 0.12 ms, *n* = 10 and 1.62 ± 0.11 ms, *n* = 10 for mean intraburst interval and mean open time, respectively, and in RT: 2.46 ± 0.12 ms, *n* = 5 and 2.20 ± 0.11 ms, *n* = 5 for mean intraburst interval and mean open time, respectively, *P* < 0.05, Fig. 4C). Both in patches from VB and in RT cells, the open time distributions had a fast and a slow component, and the respective time constants determined for these thalamic areas were not significantly different (VB: τ_fast_ = 0.49 ± 0.05 ms, and τ_slow_ = 2.18 ± 0.13 ms, *n* = 10; RT: τ_fast_ = 0.48 ± 0.06 ms and τ_slow_ = 2.57 ± 0.32 ms, *n* = 5, *P* > 0.05; Fig. 4D and E). However, the percentage of the fast component of the open time distribution was significantly larger in patches from VB cells (0.59 ± 0.04, *n* = 10, and 0.43 ± 0.05, *n* = 5 in VB and RT cells, respectively, *P* < 0.05, Fig. 4F). A similar value of the slow time constant in the open time distribution in patches from VB and RT cells suggests that a considerable proportion of channels in both cell types is characterized by a similar opening/closing kinetics. In addition, a larger proportion of the fast component in patches from VB suggests that in neurons from this nucleus, channels with brief openings are more abundant than in patches from RT.

Interestingly, as explained in detail in Barberis *et al.* (2007), the single-channel currents mediated by α1β2γ2 and α3β2γ2 receptors, recorded in analogous experimental conditions, showed similar properties. In particular, the burst duration was longer in α3β2γ2, while the opening/closing kinetics were not different between these GABA_A_R subtypes. However, the mean burst durations recorded in patches from HEK cells transfected with α1β2γ2 and α3β2γ2 cDNA were roughly half those measured in patches from neurons in VB and RT nuclei. This observation might indicate that in neurons there is a modulatory mechanism that renders the burst duration longer than in HEKs and/or that there is a contribution from different GABA_A_R subtypes (see Discussion).

### Single channels in stationary conditions

**Fig. 5:**
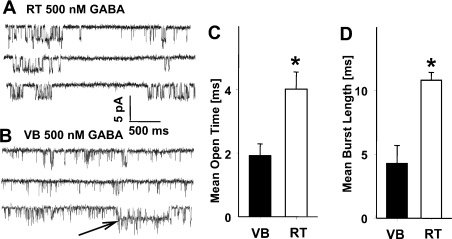
Stationary single-channel kinetics reveal substantial differences in open times and burst kinetics of channels in ventrobasal (VB) and thalamic reticular (RT) neurons. (A) and (B) Typical examples of stationary single-channel records in the presence of 500 nmγ-aminobutyric acid (GABA) in RT (A) and VB neurons (B). Note that in the example of record obtained from a VB neuron there is a long-lasting burst (arrow). (C) and (D) Summary of data for the assessment of the mean open time (C) and mean burst length (D). *P < 0.05 indicates a significant difference.

Single-channel GABAergic activity in excised patches from VB and RT neurons was also recorded in the stationary conditions (Fig. 5). In RT neurons opening bursts (Fig. 5A) appeared clearly longer than those recorded in VB neurons. However, in the steady-state conditions in VB neurons even at a GABA concentration of a few micromoles, it was difficult to collect recordings with a low percentage of overlapping single-channel events. We thus compared the single-channel stationary kinetics in VB and RT neurons at a GABA concentration of 500 nm. Apparently, at higher [GABA] we could observe qualitatively a substantial increase in burst durations (especially in RT neurons), but they could not be reliably quantified due to an excessive number of overlaps. Surprisingly, in the stationary conditions (Fig. 5C) the mean open time in RT neurons was markedly longer than in the non-stationary recordings (compare Figs 4C and 5C), while for VB neurons this difference was minor (Figs 4C and 5C). The mean burst lengths were much longer in patches from RT neurons in comparison with those from VB neurons (Fig. 5C).

Although the single-channel activity recorded from patches in VB neurons was dominated by brief openings and brief bursts, in two out of eight cells, occasionally, long bursts of activity were observed (Fig. 5B, arrow). Both brief and long openings were blocked by 25 µm bicuculline (data not shown).

## Discussion

The major finding of the present work is that the properties of GABAergic currents in VB and RT neurons largely reproduce the kinetic patterns of responses mediated by GABA_A_ receptors containing α1- and α3-subunits. Most importantly, the deactivation kinetics measured from responses to brief applications of saturating [GABA] (Fig. 2) in RT and VB neurons qualitatively mimics the proportions between decay kinetics of mIPSCs recorded in these neurons (Figs 1 and 2). Moreover, deactivation kinetics determined for current responses elicited by brief GABA applications in patches from VB and RT neurons were similar to that determined for α1β2γ2 and α3β2γ2 receptors (see exemplary traces in Fig. 2A and Barberis *et al.*, 2007 for detailed analysis of recombinant receptors). This observation suggests that in non-stationary conditions, especially in response to brief applications of high-agonist concentrations, the kinetics of GABA_A_ receptors present in these two types of neurons does indeed follow the pattern of α1- and α3-subunit-containing receptors. However, the mIPSC kinetics, both in VB and RT neurons (Fig. 1C), differs from that of current responses (Fig. 2). This difference might result from several mechanisms, e.g. electrotonic filtering, difference in GABA transient in IPSCs and in experiments with rapid responses (e.g. Mozrzymas, 2004), modulation of synaptic receptors by scaffolding proteins, e.g. gephyrin (Jacob *et al.*, 2005; van Zundert *et al.*, 2005), and differences in recording modes (mIPSCs were recorded in the whole-cell while current responses in excised patch configuration) that might differentially affect the intracellular milieu of GABA_A_Rs. A hallmark of α3-subunit-containing receptor kinetics was an extremely slow onset of currents elicited by non-saturating [GABA] that was at least one order of magnitude slower than in the case of α1β2γ2 GABA_A_R subtype (Barberis *et al.*, 2007). Importantly, this characteristic feature of α3-subunit-containing receptors was clearly reproduced in patches from RT neurons, although the averaged 10–90% rise time measured in RT neurons was slightly faster than for α3β2γ2 channels. Notably, none of the measured kinetic parameters of current responses showed as large a difference between VB and RT neurons (and between currents mediated by α1β2γ2 and α3β2γ2 receptors). These observations together with a substantial variability of the onset kinetics in RT neurons (for non-saturating [GABA]) suggest that although α3-subunit-containing receptors play a major role in determining the kinetic behavior of GABAergic currents in patches from RT neurons, probably also other receptors could be involved. Indeed, while our data clearly indicate that in VB and RT nuclei the non-stationary GABAergic currents follow the kinetic pattern of responses mediated by α1- and α3-subunit-containing receptors, the time course of these currents might additionally depend on β-subunit type. Huntsman & Huguenard (2000) have suggested that the β3-subunit was predominant in RT neurons and, more recently, a contribution of the β1-subunit in this nucleus has also been implicated (Huntsman & Huguenard, 2006). Thus, a variety of β-subunits in VB and RT neurons might underlie, at least partially, the differences between currents recorded from these neurons and those mediated by α1β2γ2 and α3β2γ2 receptors. A thorough assessment of the impact of various β-subunits on currents mediated by α1- and α3-subunit-containing receptors would require further extensive studies based on analysis of high-resolution recordings similar to those employed in our recent study (Barberis *et al.*, 2007).

The notion that the kinetics of non-stationary current responses in VB and RT neurons are similar to that mediated by α1- and α3-subunit-containing receptors is further supported by the analysis of single-channel kinetics during the deactivation phase (Fig. 4). In these conditions, similarly to recombinant α1β2γ2 and α3β2γ2 receptors, channels in VB and RT neurons appeared to have a similar opening/closing kinetics, although in VB there was a slightly larger percentage of brief openings and the mean open time in this thalamic region was a little faster (Fig. 4C and F). Such a contribution of short-lasting channel activity could be due to receptors containing the δ-subunit (Laurie *et al.*, 1992; Fisher & Macdonald, 1997; Jia *et al.*, 2005; Chandra *et al.*, 2006; Kralic *et al.*, 2006; Ortinski *et al.*, 2006). In our previous published work (Ortinski *et al.*, 2006), we compared single-channel recordings in patches excised from cerebellar granule neurons from +/+ and α1 –/– mice, and not δ–/–. Because in these neurons α1βγ2 and α6βδ receptors are the two major subtypes of synaptic and extrasynaptic receptors, we could compare the characteristics of single-channel currents with those reported for δ-containing channels (Fisher & Macdonald, 1997). From this study it is clear that the two major characteristics of δ-containing channels are a similar conductance to γ-containing ones and very brief open time lacking longer burst openings. Unfortunately, these characteristics are shared by α1βγ2 when activated by low-agonist concentrations, as shown in Barberis *et al.* (2007), precluding the possibility to distinguish them in patches from neurons containing both channel types. Indeed, in Ortinski *et al.* (2006), removal of the α1-subunit lead to the disappearance of the relatively small proportion of longer burst seen in +/+ patches, but did not substantially change the characteristics of the predominant brief openings. Activation of channels with a low concentration of THIP (4,5,6,7-tetrahydroisoxazolo[5,4-c]pyridin-3-ol) also lead to predominant brief openings, although with higher opening frequency as expected (our unpublished results).

While in VB and RT neurons the kinetic properties of currents elicited by brief applications of high [GABA] closely resembled currents mediated by α1- and α3-subunit-containing receptors, in the steady-state conditions clear differences between neuronal and respective recombinant GABA_A_Rs were seen (Fig. 5, Barberis *et al.*, 2007). Most importantly, when continuously exposed to low (500 nm) GABA concentration, the mean open time for GABA_A_Rs in patches from RT neurons was markedly longer than in those from VB suggesting that, in these conditions, opening/closing kinetics in these receptors are different. The reason for this discrepancy is not clear, although there are several likely explanations that need to be considered. As already mentioned above, VB neurons are rich in δ-subunit-containing GABA_A_Rs. These receptors are endowed with high affinity to GABA and relatively slow kinetics. Thus, it is possible that the extent of their activation during a short and saturating GABA pulse would be small, while in the presence of a low but continuously applied [GABA] their contribution to the overall activity would be considerably larger. Moreover, the kinetic behavior of a GABA_A_R subtype does not depend solely on the subunit composition, as it can be modulated by a variety of intracellular messengers. For instance, we have shown that α1β2γ2 receptors encoded by exactly the same plasmids but expressed in different cell lines (HEK293 and QT-6) showed a clear difference in their kinetics (Mercik *et al.*, 2003). It is thus possible that differences in burst durations in non-stationary conditions between neuronal and respective recombinant receptors might be, at least in part, due to an unknown modulatory mechanism that is present in neurons and absent in HEK293 cells (or vice versa). Our observation regarding the difference between stationary open kinetics in VB and RT neurons is in agreement with that reported by Browne *et al.* (2001).

Although in RT neurons bursts were clearly longer and more frequent than in VB regions, in both neuronal types a considerable percentage of single brief events was detected (Fig. 5A and B). It should be emphasized, however, that stationary recordings that were included in our quantitative analysis were measured at low (500 nm) GABA concentration (to reduce the occurrence of overlapping events). It is thus likely that brief and single openings in RT neurons could be due to singly bound receptors. Macdonald *et al.* (1989) have suggested that, at micromolar [GABA], a considerable proportion of single-channel activity was due to singly bound GABA_A_Rs. Moreover, α3-subunit-containing receptors, that are likely to be predominant in RT neurons (Laurie *et al.*, 1992; Pirker *et al.*, 2000; Kralic *et al.*, 2006), are known to show a relatively low affinity, suggesting that the percentage of singly bound receptors at 500 nm GABA might be higher in RT than in VB neurons, where GABA_A_Rs are endowed with higher affinity. Thus, the fact that, in spite of a probable larger contribution of singly bound receptors, averaged bursts are longer in RT neurons further suggests that in this nucleus the bursting mode of single-channel activity is more pronounced than in VB neurons. On the other hand, according to a recent report by Huntsman & Huguenard (2006), in RT neurons there could be a subpopulation of β1-subunit-containing receptors characterized by a relatively fast gating and giving rise to a fast mIPSC component in this nucleus. Altogether, our single-channel data showed that GABA_A_Rs in RT neurons are characterized by longer bursts than in VB nucleus but, as already mentioned, this cannot exhaustively explain profound differences in mIPSC kinetics observed in VB and RT neurons. While our non-stationary data (Fig. 4) closely resembled those obtained for recombinant α1- and α3-subunit-containing receptors (Barberis *et al.*, 2007), steady-state recordings revealed a variety of kinetic patterns and showed substantial differences with respect to these recombinant receptors. This might suggest that receptors responding to brief (synaptic-like) agonist pulses may show a marked degree of functional and/or structural homogeneity, while those exposed to tonic stimuli show clear functional differences suggesting involvement of different receptor subtypes.

As already mentioned in the Results, the single-channel steady-state kinetics measured from VB neurons could not be ascribed to an entirely homogeneous receptor population, as in two out of eight cells the occurrence of long bicuculline-sensitive bursts (similar to those observed in RT neurons) was observed. The identity of GABA_A_Rs involved in generating this form of single-channel activity is not known. Altogether, it is worth emphasizing that single-channel activity measured in both thalamic areas might show detectable contributions of both brief events and long bursts, but the proportion of the latter is consistently larger in RT neurons while the former appear to predominate in VB neurons (Browne *et al.*, 2001).

The physiological role of kinetic differences between IPSCs generated by VB and RT neurons is not clear. RT nucleus is particularly abundant in GABAergic interneurons that send a potent inhibitory output from this nucleus. It seems that unusually long-lasting IPSC kinetics in these neurons provides a particularly potent mechanism for inhibitory drive. We may speculate that such a slow IPSC kinetics in RT indicates that the mechanisms controlling these inhibitory interneurons do not require a high temporal precision but rather rely on a more sustained action. This possibility is supported by the observation that IPSCs in RT neurons do not undergo a typically observed developmental speed up, and the expression of α3-subunit containing neurons is maintained throughout development and adulthood (Laurie *et al.*, 1992; Pirker *et al.*, 2000; Kralic *et al.*, 2006). Moreover, the fact that GABA_A_Rs in RT neurons respond very slowly to non-saturating [GABA] (Fig. 3) further indicates that their kinetic ‘design’ makes them most suitable to mediate slow and sustained signals. In contrast, IPSCs in VB are much faster than in RT neurons and undergo further speed up during development, making them more appropriate for tasks requiring a high temporal resolution (e.g. coincidence detection of synaptic currents). However, high expression of δ-subunit and frequent occurrence of brief events at low [GABA] further support that VB neurons, besides fast IPSCs, are endowed with a more potent tonic inhibition than RT neurons (Jia *et al.*, 2005; Bellelli *et al.*, 2005; Cope *et al.*, 2005; Chandra *et al.*, 2006).

## Abbreviations

GABA: γ-aminobutyric acid
GABA_A_Rs: GABA_A_ receptors
HEK: human embryonic kidney
IPSC: inhibitory postsynaptic current
P: postnatal day
RT: thalamic reticular
VB: ventrobasal
